# Supplementary material for: GLH/VASA helicases promote germ granule formation to ensure the fidelity of piRNA-mediated transcriptome surveillance
Source: Nat Commun. 2022 Sep 9;13:5306. doi: 10.1038/s41467-022-32880-2 (PMC9463143; doi:10.1038/s41467-022-32880-2)
Supplement: Supplementary file 1 — Supplementary Information File [file 41467_2022_32880_MOESM1_ESM.pdf]

GLH/VASA helicases promote germ granule formation to ensure the fidelity of piRNA-mediated transcriptome surveillance

Wenjun Chen[1, 2†], Jordan S Brown[1†], Tao He[3], Wei-Sheng Wu[4], Shikui Tu[5], Zhiping Weng[6], Donglei Zhang[2*], and Heng-Chi Lee[1*]

Supplementary Information

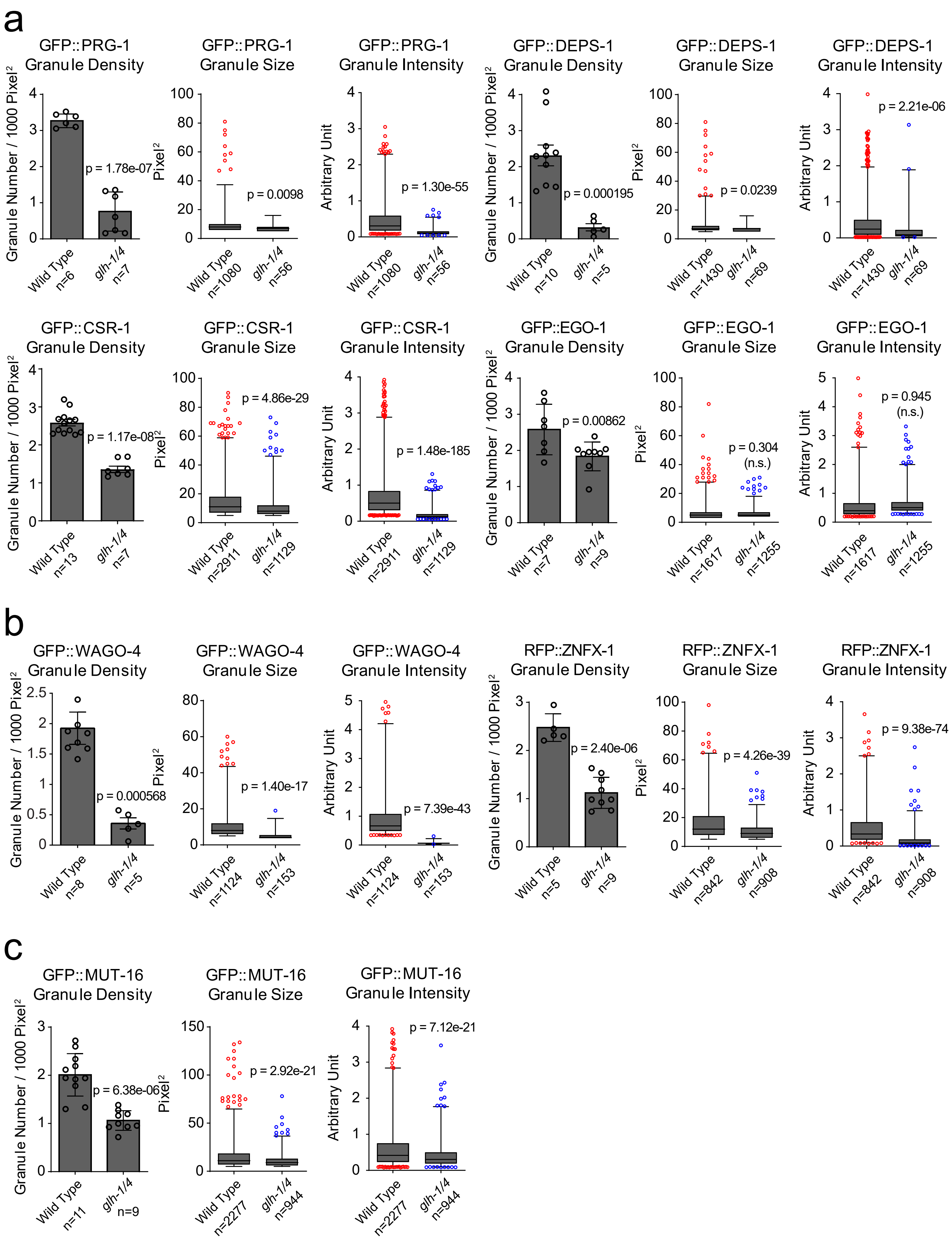
a
GFP::PRG-1 Granule Density
Granule Number / 1000 Pixel²
p = 1.78e-07
Wild Type n=6
glh-1/4 n=7
GFP::PRG-1 Granule Size
Pixel²
p = 0.0098
Wild Type n=1080
glh-1/4 n=56
GFP::PRG-1 Granule Intensity
Arbitrary Unit
p = 1.30e-55
Wild Type n=1080
glh-1/4 n=56
GFP::DEPS-1 Granule Density
Granule Number / 1000 Pixel²
p = 0.000195
Wild Type n=10
glh-1/4 n=5
GFP::DEPS-1 Granule Size
Pixel²
p = 0.0239
Wild Type n=1430
glh-1/4 n=69
GFP::DEPS-1 Granule Intensity
Arbitrary Unit
p = 2.21e-06
Wild Type n=1430
glh-1/4 n=69
GFP::CSR-1 Granule Density
Granule Number / 1000 Pixel²
p = 1.17e-08
Wild Type n=13
glh-1/4 n=7
GFP::CSR-1 Granule Size
Pixel²
p = 4.86e-29
Wild Type n=2911
glh-1/4 n=1129
GFP::CSR-1 Granule Intensity
Arbitrary Unit
p = 1.48e-185
Wild Type n=2911
glh-1/4 n=1129
GFP::EGO-1 Granule Density
Granule Number / 1000 Pixel²
p = 0.00862
Wild Type n=7
glh-1/4 n=9
GFP::EGO-1 Granule Size
Pixel²
p = 0.304 (n.s.)
Wild Type n=1617
glh-1/4 n=1255
GFP::EGO-1 Granule Intensity
Arbitrary Unit
p = 0.945 (n.s.)
Wild Type n=1617
glh-1/4 n=1255
b
GFP::WAGO-4 Granule Density
Granule Number / 1000 Pixel²
p = 0.000568
Wild Type n=8
glh-1/4 n=5
GFP::WAGO-4 Granule Size
Pixel²
p = 1.40e-17
Wild Type n=1124
glh-1/4 n=153
GFP::WAGO-4 Granule Intensity
Arbitrary Unit
p = 7.39e-43
Wild Type n=1124
glh-1/4 n=153
RFP::ZNFX-1 Granule Density
Granule Number / 1000 Pixel²
p = 2.40e-06
Wild Type n=5
glh-1/4 n=9
RFP::ZNFX-1 Granule Size
Pixel²
p = 4.26e-39
Wild Type n=842
glh-1/4 n=908
RFP::ZNFX-1 Granule Intensity
Arbitrary Unit
p = 9.38e-74
Wild Type n=842
glh-1/4 n=908
c
GFP::MUT-16 Granule Density
Granule Number / 1000 Pixel²
p = 6.38e-06
Wild Type n=11
glh-1/4 n=9
GFP::MUT-16 Granule Size
Pixel²
p = 2.92e-21
Wild Type n=2277
glh-1/4 n=944
GFP::MUT-16 Granule Intensity
Arbitrary Unit
p = 7.12e-21
Wild Type n=2277
glh-1/4 n=944

Supplementary Fig. 1. Image analyses corresponding to Figure 1A-C

a) Granule density, size, and intensity quantification of proteins known to be enriched in P granules in the wild type or the *glh-1 glh-4* mutant, corresponding to micrographs in Figure 1b. Statistical analysis was performed using a one-tailed Student's t-test. Bars indicate the mean, errors bars indicate the standard deviation, and data points indicate values for individual worms (density) or granules (size and intensity) from each genotype. N values indicate number of worms (density) or granules (size and intensity) examined.
b) Granule density, size, and intensity quantification of proteins known to be enriched in Z granules in the wild type or the *glh-1 glh-4* mutant, corresponding to micrographs in Figure 1c. Statistical analysis was performed using a one-tailed Student's t-test. Bars indicate the mean, errors bars indicate the standard deviation, and data points indicate values for individual worms (density) or granules (size and intensity) from each genotype. N values indicate number of worms (density) or granules (size and intensity) examined.
c) Granule density, size, and intensity quantification of proteins known to be enriched in mutator granules in the wild type or the *glh-1 glh-4* mutant, corresponding to micrographs in Figure 1d. Each bar represents n = 8~10 worms. Statistical analysis was performed using a one-tailed Student's t-test. Bars indicate the mean, errors bars indicate the standard deviation, and data points indicate values for individual worms (density) or granules (size and intensity) from each genotype. N values indicate number of worms (density) or granules (size and intensity) examined.

a

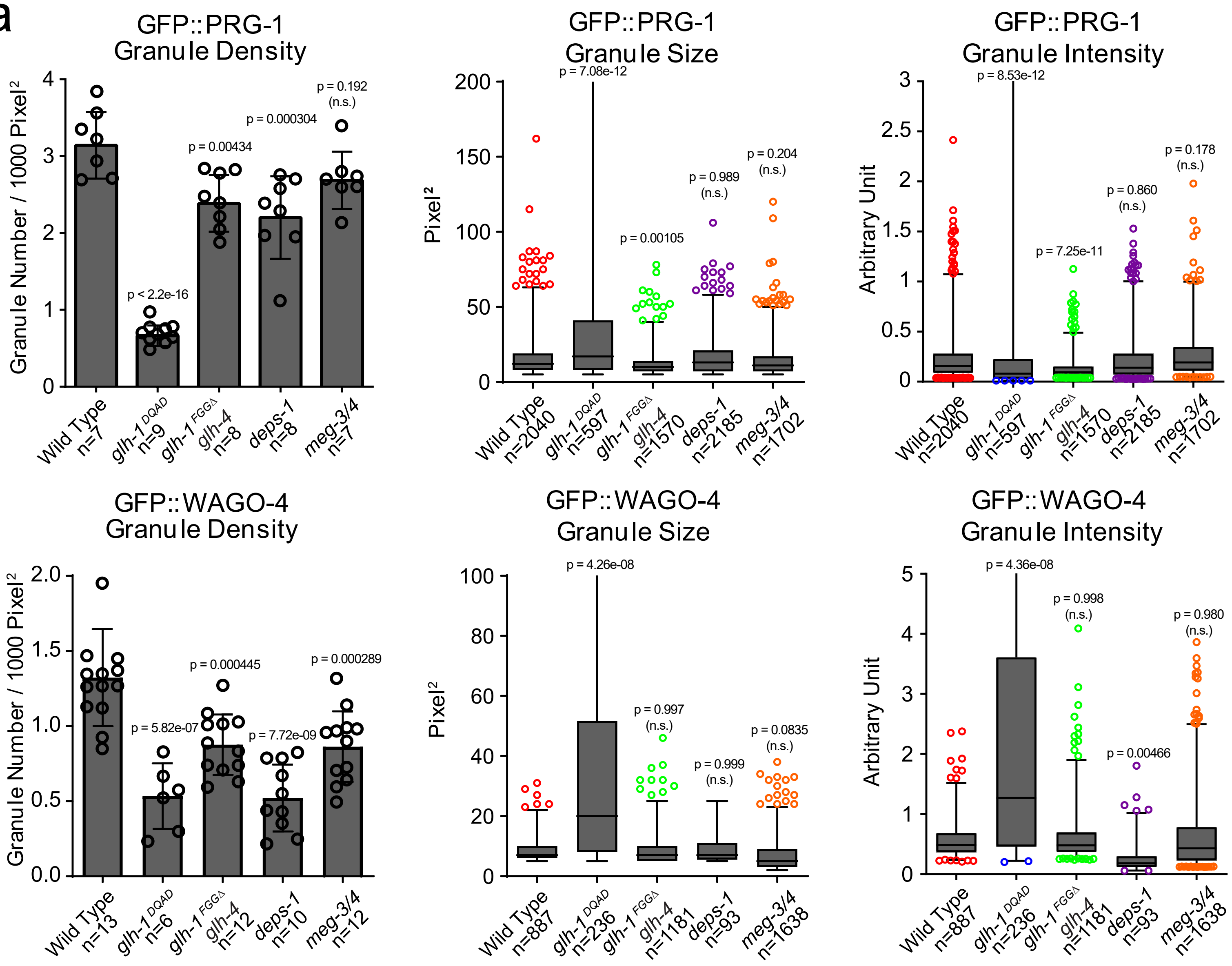

GFP::PRG-1 Granule Density
Granule Number / 1000 Pixel²
p < 2.2e-16
p = 0.00434
p = 0.000304
p = 0.192 (n.s.)
Wild Type n=7
glh-1 DQAD n=9
glh-1 FGGΔ
glh-4 n=8
deps-1 n=8
meg-3/4 n=7
GFP::PRG-1 Granule Size
Pixel²
p = 7.08e-12
p = 0.00105
p = 0.989 (n.s.)
p = 0.204 (n.s.)
Wild Type n=2040
glh-1 DQAD n=597
glh-1 FGGΔ
glh-4 n=1570
deps-1 n=2185
meg-3/4 n=1702
GFP::PRG-1 Granule Intensity
Arbitrary Unit
p = 8.53e-12
p = 7.25e-11
p = 0.860 (n.s.)
p = 0.178 (n.s.)
GFP::WAGO-4 Granule Density
Granule Number / 1000 Pixel²
p = 5.82e-07
p = 0.000445
p = 7.72e-09
p = 0.000289
Wild Type n=13
glh-1 DQAD n=6
glh-1 FGGΔ
glh-4 n=12
deps-1 n=10
meg-3/4 n=12
GFP::WAGO-4 Granule Size
Pixel²
p = 4.26e-08
p = 0.997 (n.s.)
p = 0.999 (n.s.)
p = 0.0835 (n.s.)
Wild Type n=887
glh-1 DQAD n=236
glh-1 FGGΔ
glh-4 n=1181
deps-1 n=93
meg-3/4 n=1638
GFP::WAGO-4 Granule Intensity
Arbitrary Unit
p = 4.36e-08
p = 0.998 (n.s.)
p = 0.00466
p = 0.980 (n.s.)

b

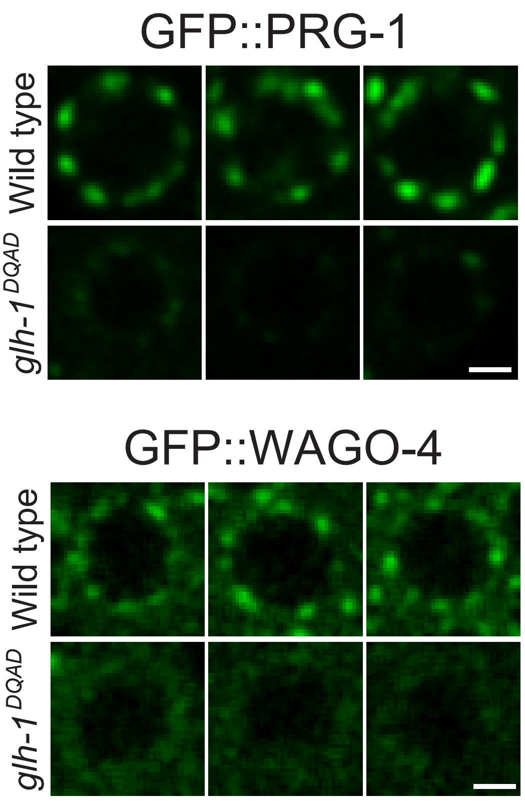

GFP::PRG-1
Wild type
glh-1 DQAD
GFP::WAGO-4
Wild type
glh-1 DQAD

C

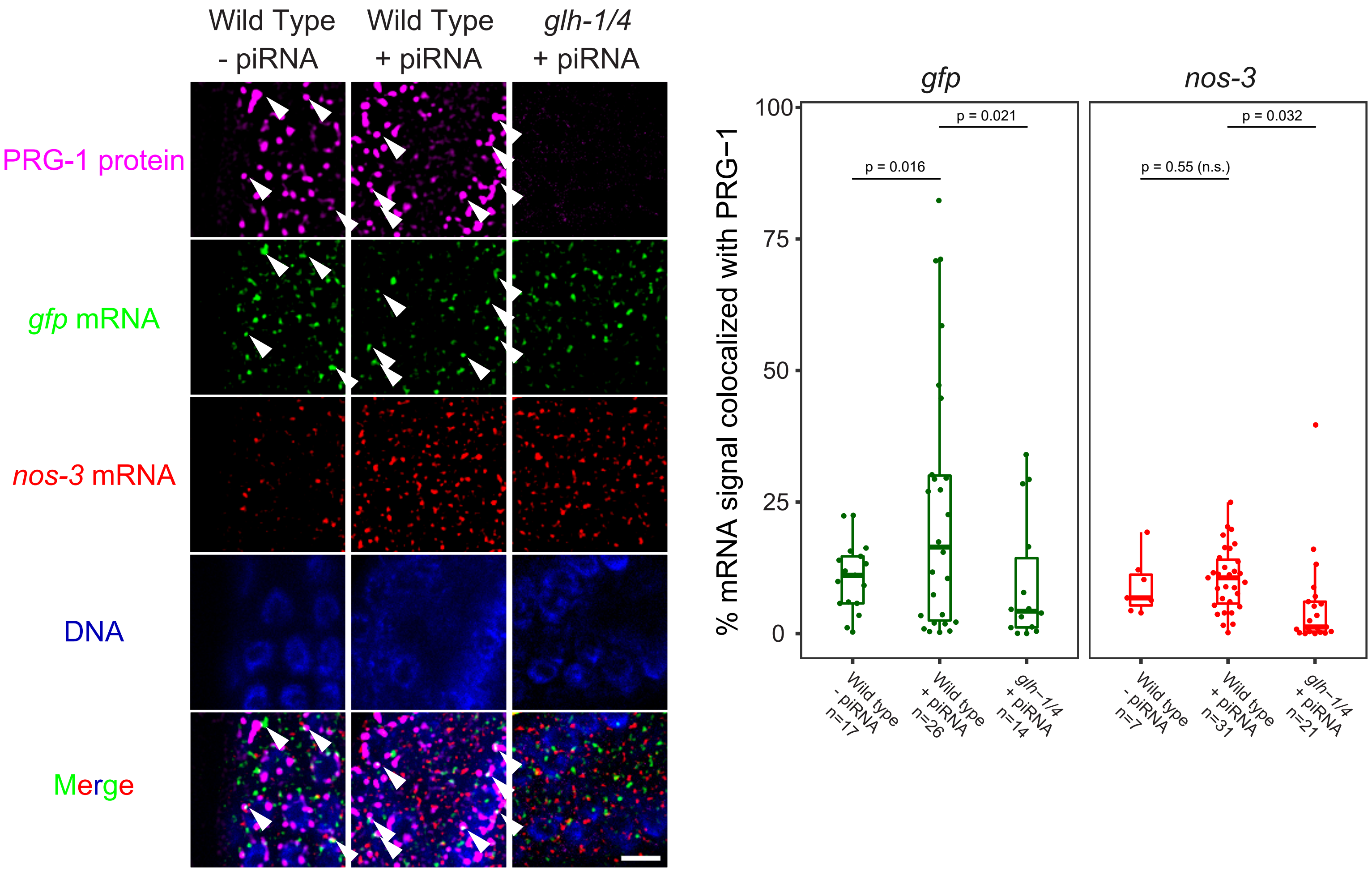

Wild Type
- piRNA
Wild Type
+ piRNA
glh-1/4
+ piRNA
PRG-1 protein
gfp mRNA
nos-3 mRNA
DNA
Merge
gfp
nos-3
% mRNA signal colocalized with PRG−1
100
75
50
25
0
p = 0.016
p = 0.021
p = 0.55 (n.s.)
p = 0.032
Wild type
- piRNA
n=17
Wild type
+ piRNA
n=26
glh−1/4
+ piRNA
n=14
Wild type
- piRNA
n=7
Wild type
+ piRNA
n=31
glh−1/4
+ piRNA
n=21

Supplementary Fig. 2. Image analyses corresponding to Figure 2B

a) Granule density, size, and intensity quantification of GFP::PRG-1 and GFP::WAGO-4 in the indicated genotypes, corresponding to micrographs in Figure 2b and 3a. Statistical analysis was performed using a one-way ANOVA and Tukey's correction for multiple comparisons were used. Bars indicate the mean, errors bars indicate the standard deviation, and data points indicate values for individual worms (density) or granules (size and intensity) from each genotype. N values indicate number of worms (density) or granules (size and intensity) examined.

b) Single nuclei from micrographs presented in Figure 2b. Bar, 2 micrometers.

c) Colocalization analysis of mRNA signal (*gfp* and *nos-3*) with PRG-1 signal. An example single z-slice image from each genotype is presented to the left. Arrowheads indicate pixels showing *gfp* mRNA signal colocalizing with PRG-1 signal. Colocalized pixels are highlighted in white in the merged image. Bar, 5 micrometers. Quantification is shown to the right. Each dot represents a z-series from a single gonad of the indicated genotype, probed under the indicated conditions. Data points represent biological replicates in two independent experiments. Statistical analysis was performed using a two-tailed Student's t-test. For all boxplots, lines display median values, boxes display first and third quartiles, and whiskers display 5th and 95th percentiles.

a

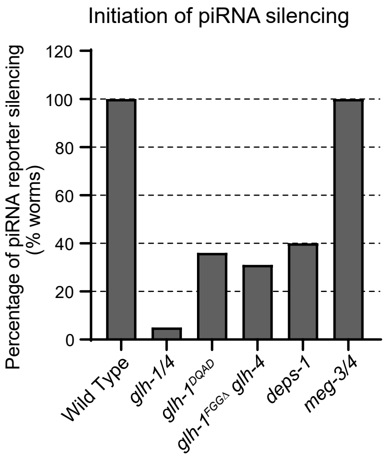
Initiation of piRNA silencing
Percentage of piRNA reporter silencing (% worms)
120
100
80
60
40
20
0
Wild Type
glh-1/4
glh-1DQAD
glh-1FGGΔ glh-4
deps-1
meg-3/4

Supplementary Fig. 3. Mutants with defects in PRG-1 perinuclear condensates exhibit defects in triggering piRNA silencing

a) The percentage of F2 generation worms that exhibit silencing of a GFP transgene in the indicated strains. For *meg-3 meg-4* mutants, n = 14. For all other strains, n > 50.

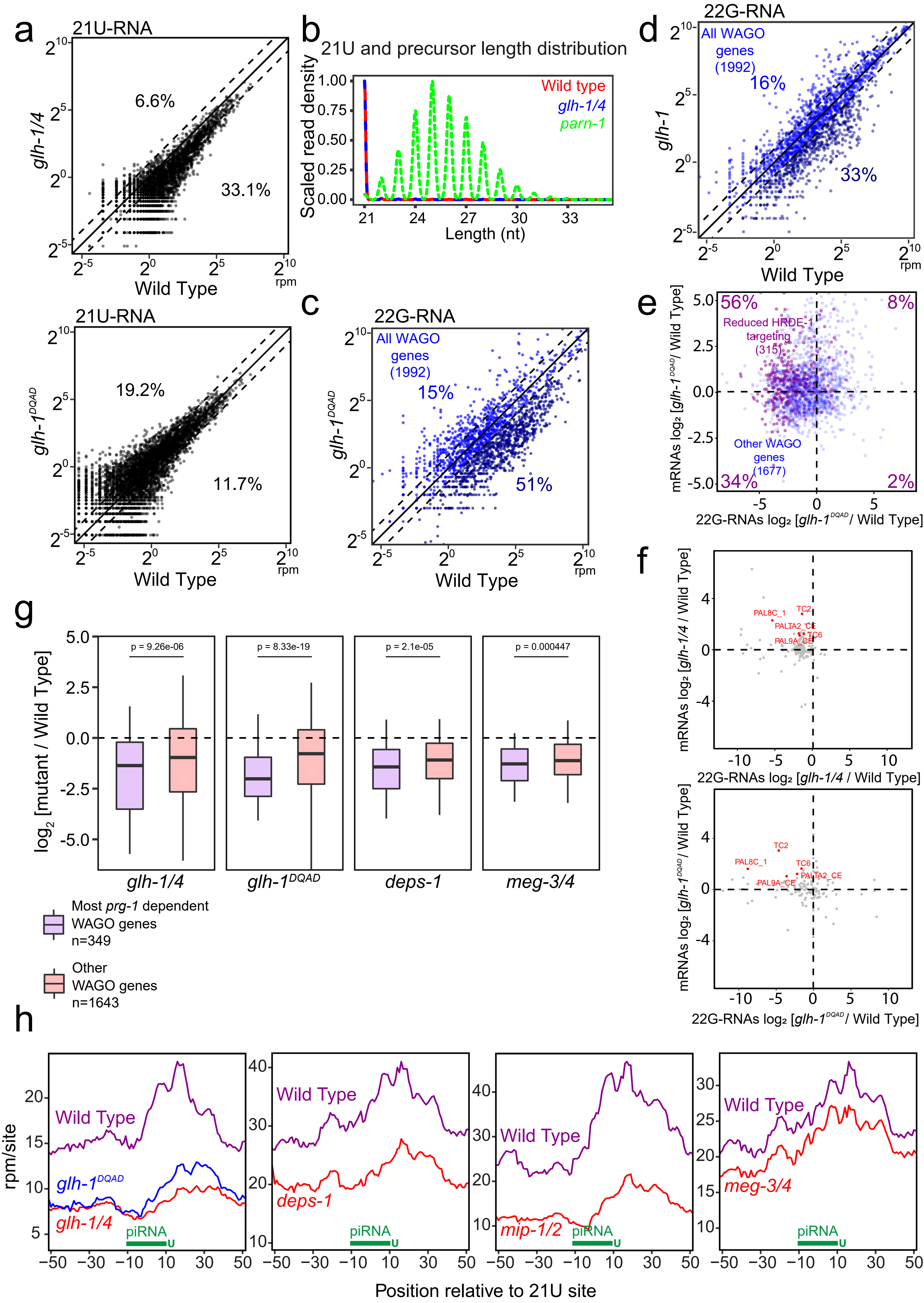

a
21U-RNA
glh-1/4
Wild Type
6.6%
33.1%
rpm
b
21U and precursor length distribution
Scaled read density
Length (nt)
Wild type
glh-1/4
parn-1
d
22G-RNA
All WAGO genes (1992)
16%
33%
glh-1
Wild Type
rpm
21U-RNA
glh-1DQAD
19.2%
11.7%
Wild Type
rpm
c
22G-RNA
All WAGO genes (1992)
15%
51%
glh-1DQAD
Wild Type
rpm
e
56%
8%
34%
2%
Reduced HRDE-1 targeting (315)
Other WAGO genes (1677)
mRNAs log2 [glh-1DQAD / Wild Type]
22G-RNAs log2 [glh-1DQAD / Wild Type]
f
mRNAs log2 [glh-1/4 / Wild Type]
22G-RNAs log2 [glh-1/4 / Wild Type]
PAL8C_1
TC2
PALTA2_CE
TC6
PAL9A_CE
mRNAs log2 [glh-1DQAD / Wild Type]
22G-RNAs log2 [glh-1DQAD / Wild Type]
g
log2 [mutant / Wild Type]
p = 9.26e-06
p = 8.33e-19
p = 2.1e-05
p = 0.000447
glh-1/4
glh-1DQAD
deps-1
meg-3/4
Most prg-1 dependent WAGO genes n=349
Other WAGO genes n=1643
h
rpm/site
Wild Type
glh-1DQAD
glh-1/4
deps-1
mip-1/2
meg-3/4
piRNA
U
Position relative to 21U site

Supplementary Fig. 4. GLH/VASA mutants exhibit defects in producing secondary WAGO-22G-RNAs (Corresponding to Figure 4)

a) A scatter plot showing the abundance of each piRNA (21U-RNA) in *glh-1 glh-4* and *glh-1 DQAD* mutant worms compared to wild type. Diagonal lines indicate a two-fold increase (top), no change (middle), or a two-fold depletion (bottom) in the indicated mutant strains. Percentages indicate the proportion of piRNAs increased or decreased 2-fold compared to wild type.

b) Scaled kernel density estimation of mature piRNAs (21nt) and their precursors (>21nt) in the indicated strains.

c-d) Scatter plots showing the abundance of all 22G-RNAs mapped to each WAGO targets in wild type worms compared to the indicated mutants. The percentage of WAGO targets with 2-fold increased or decreased 22G-RNAs in mutants are shown. Diagonal lines indicate a two-fold increase (top), no change (middle), or a two-fold depletion (bottom) in the mutant.

e) A scatter plot showing the mRNA vs 22G-RNA expression changes for WAGO targets in *glh-1 DQAD* mutants vs wild type worms. Percentages indicate the proportion of WAGO targets with reduced HRDE-1-associated 22G-RNAs that fall in that quadrant.

f) A scatter plot showing the mRNA vs 22G-RNA expression changes for transposon mRNA in the indicated mutants vs wild type worms. Transposons with increased mRNAs and decreased 22G-RNAs in both *glh-1 glh-4* and *glh-1 DQAD* mutants are highlighted in red. 22G-RNA data reflect data collected from a single experiment. mRNA data reflect data collected across 2 biological replicates.

g) 22G-RNA fold changes for WAGO targets most dependent on *prg-1* for 22G-RNA accumulation in the indicated mutants compared to wild type. Most *prg-1* dependent WAGO targets defined as those WAGO targets with a significant (adjusted $P < 0.05$ [see Methods for details] and 2-fold) decrease in 22G-RNA accumulation in *prg-1* mutants. Statistical analysis was performed using a two-tailed Mann-Whitney Wilcoxon test. For all boxplots, lines display median values, boxes display first and third quartiles, and whiskers display 5th and 95th percentiles.

h) Density of 22G-RNAs within a 100 nt window around predicted piRNA target sites in the indicated strains. Computed by summing 22G-RNA density per piRNA targeting site in all WAGO targets. Plots are centered on the 10th nucleotide of piRNAs.

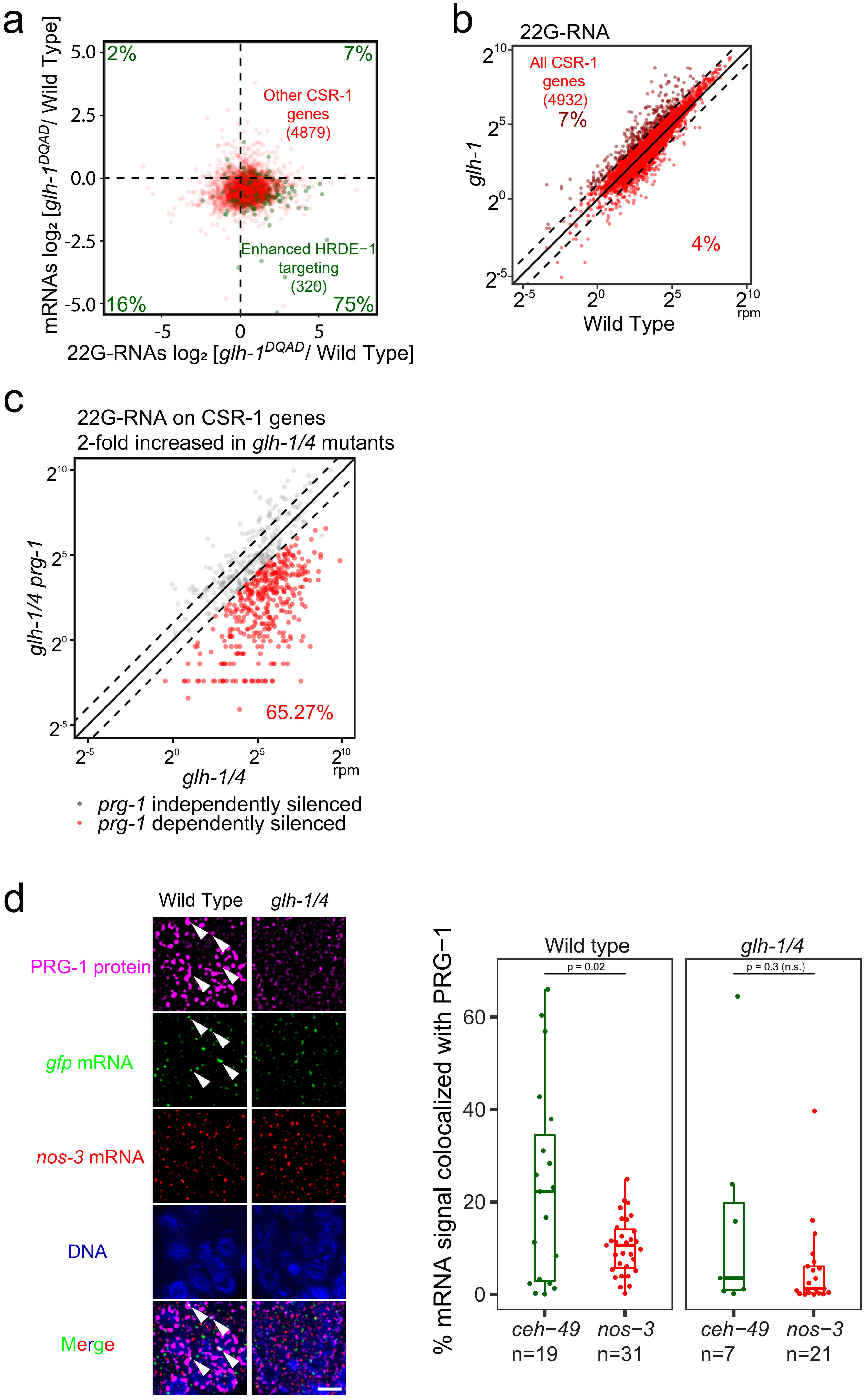
a
mRNAs log₂ [glh-1DQAD/ Wild Type]
22G-RNAs log₂ [glh-1DQAD/ Wild Type]
2%
7%
16%
75%
Other CSR-1 genes (4879)
Enhanced HRDE−1 targeting (320)
b
22G-RNA
All CSR-1 genes (4932)
7%
4%
glh-1
Wild Type
rpm
c
22G-RNA on CSR-1 genes
2-fold increased in glh-1/4 mutants
glh-1/4 prg-1
glh-1/4
rpm
65.27%
prg-1 independently silenced
prg-1 dependently silenced
d
Wild Type
glh-1/4
PRG-1 protein
gfp mRNA
nos-3 mRNA
DNA
Merge
% mRNA signal colocalized with PRG−1
Wild type
glh-1/4
p = 0.02
p = 0.3 (n.s.)
ceh−49
n=19
nos−3
n=31
ceh−49
n=7
nos−3
n=21

Supplementary Fig. 5. Many functional germline genes are silenced in mutants with defects in forming perinuclear PRG-1 granules (Corresponding to Figure 5)

a) A scatter plot showing the mRNA vs 22G-RNA $\log_2$ expression changes for CSR-1 targets in *glh-1 DQAD* mutants vs wild type worms. The bottom right quadrant corresponds to CSR-1 targets that have become silenced in the mutant (decreased mRNAs and increased 22G-RNAs). Percentages are shown in each quadrant to indicate the proportion of CSR-1 targets with enhanced HRDE-1-associated 22G-RNAs in the *glh-1* mutant that fall in that quadrant.

b) A scatter plot showing the abundance of all 22G-RNAs mapped to each CSR-1 targets in wild type worms compared to *glh-1* mutants. The percentage of CSR-1 targets with 2-fold increased or decreased 22G-RNAs in mutants are shown. The three diagonal lines indicate a two-fold increase (top), no change (middle), or a two-fold depletion (bottom) in *glh-1* mutants.

c) A scatter plot showing 22G-RNA accumulation on CSR-1 genes with a 2-fold increase in 22G-RNA accumulation in *glh-1 glh-4* mutants. The *glh-1 glh-4 prg-1* triple mutant is compared to the *glh-1 glh-4* double mutant. In red, enhanced CSR-1 targets that are suppressed by *prg-1* loss are shown. The three diagonal lines indicate a two-fold increase (top), no change (middle), or a two-fold depletion (bottom) in the indicated mutant strains. Distributions represent data collected across 2 biological replicates.

d) Colocalization analysis of mRNA signal (*ceh-49* and *nos-3*) with PRG-1 signal. An example single z-slice image from each genotype is presented to the left. Arrowheads indicate pixels showing *ceh-49* mRNA signal colocalizing

with PRG-1 signal. Colocalized pixels are highlighted in white in the merged image. Bar, 5 micrometers. Quantification is shown to the right. Each dot represents a z-series from a single gonad of the indicated genotype, probed under the indicated conditions. Data points represent biological replicates in two independent experiments. Statistical analysis was performed using a two-tailed Student's t-test. For all boxplots, lines display median values, boxes display first and third quartiles, and whiskers display 5th and 95th percentiles.

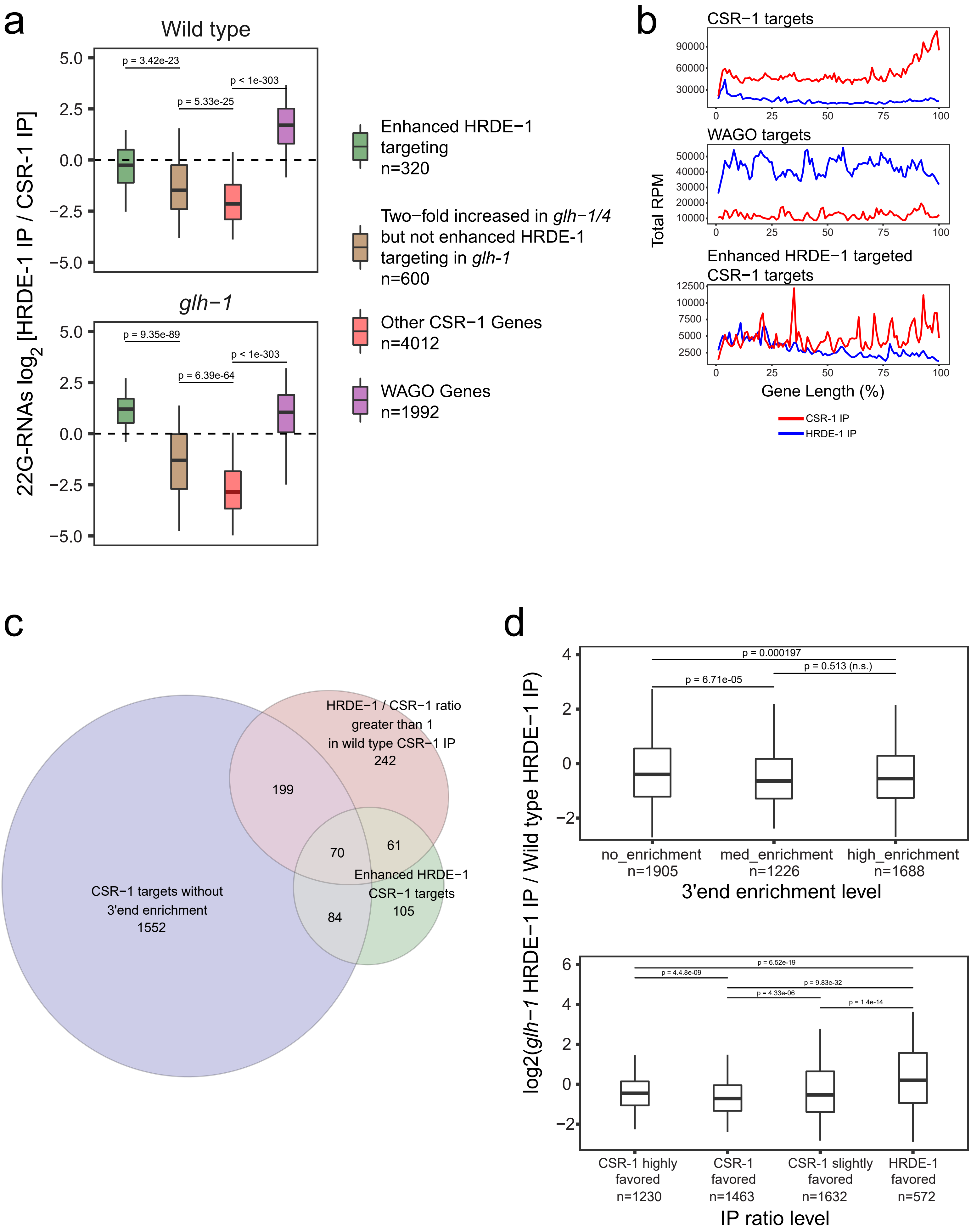

a
Wild type
p = 3.42e-23
p = 5.33e-25
p < 1e-303
glh−1
p = 9.35e-89
p = 6.39e-64
p < 1e-303
22G-RNAs log2 [HRDE-1 IP / CSR-1 IP]
Enhanced HRDE−1 targeting n=320
Two−fold increased in glh−1/4 but not enhanced HRDE-1 targeting in glh-1 n=600
Other CSR−1 Genes n=4012
WAGO Genes n=1992
b
CSR−1 targets
WAGO targets
Enhanced HRDE−1 targeted CSR−1 targets
Total RPM
Gene Length (%)
CSR-1 IP
HRDE-1 IP
c
HRDE−1 / CSR−1 ratio greater than 1 in wild type CSR−1 IP 242
199
70
61
CSR−1 targets without 3'end enrichment 1552
84
Enhanced HRDE−1 CSR−1 targets 105
d
p = 0.000197
p = 0.513 (n.s.)
p = 6.71e-05
no_enrichment n=1905
med_enrichment n=1226
high_enrichment n=1688
3'end enrichment level
p = 6.52e-19
p = 4.4.8e-09
p = 9.83e-32
p = 4.33e-06
p = 1.4e-14
log2(glh−1 HRDE−1 IP / Wild type HRDE−1 IP)
CSR-1 highly favored n=1230
CSR-1 favored n=1463
CSR-1 slightly favored n=1632
HRDE-1 favored n=572
IP ratio level

Supplementary Fig. 6. HRDE-1 / CSR-1 IP ratio and CSR-1 IP 3' end enrichment are both associated with enhanced HRDE-1 targeting of CSR-1 genes in VASA mutants.

a) 22G-RNA enrichment in HRDE-1 versus CSR-1 IP experiments in the indicated strains for CSR-1 targets and WAGO targets. Dotted line indicates no enrichment for either Argonaute. Statistical analysis was performed using a two-tailed Mann-Whitney Wilcoxon test. For all boxplots, lines display median values, boxes display first and third quartiles, and whiskers display 5th and 95th percentiles. Distributions represent data collected across 2 biological replicates for CSR-1 IP and a single experiment for HRDE-1 IP.

b) Metagene traces showing the total accumulation of 22G-RNAs by percentage of gene length in IP experiments. Traces are shown for three classes of genes: all CSR-1 targets (left), all WAGO targets (middle), and CSR-1 targets with enhanced HRDE-1 targeting (right). Traces from CSR-1 IP experiments are shown in red and traces from HRDE-1 IP experiments are shown in blue.

c) Venn diagram showing the overlap between CSR-1 targets that fail to show a 3' end enrichment in wild type CSR-1 IP (see Methods), CSR-1 targets with a HRDE-1 to CSR-1 IP ratio greater than 1, and CSR-1 targets with enhanced HRDE-1 targeting in *glh-1* mutants.

d) Characterization of the effect of CSR-1 22G-RNA 3' end enrichment on the level of HRDE-1 enhancement in *glh-1* mutants (top), and the effect of HRDE-1 to CSR-1 IP ratio on the level of HRDE-1 enhancement in *glh-1* mutants (bottom). For 3' end enrichment and HRDE-1 to CSR-1 IP ratio characterization, see Methods. Statistical analysis was performed using a two-tailed Mann-Whitney Wilcoxon test. For all boxplots, lines display median

values, boxes display first and third quartiles, and whiskers display 5th and 95th percentiles. Distributions represent data collected across 2 biological replicates for CSR-1 IP derived values and a single experiment for HRDE-1 IP derived values.
