# Supplementary material for: GLH/VASA helicases promote germ granule formation to ensure the fidelity of piRNA-mediated transcriptome surveillance
Source: Nat Commun. 2022 Sep 9;13:5306. doi: 10.1038/s41467-022-32880-2 (PMC9463143; doi:10.1038/s41467-022-32880-2)
Supplement: Supplementary file 3 — Description of Additional Supplementary Files [file 41467_2022_32880_MOESM3_ESM.pdf]

Description of Additional Supplementary Files

File Name: Supplementary Data 1
Description: List of interacting proteins identified by MS with DTME-crosslinking using GLH-1.

File Name: Supplementary Data 2
Description: List of smFISH probes.
