# Supplementary material for: GLH/VASA helicases promote germ granule formation to ensure the fidelity of piRNA-mediated transcriptome surveillance
Source: Nat Commun. 2022 Sep 9;13:5306. doi: 10.1038/s41467-022-32880-2 (PMC9463143; doi:10.1038/s41467-022-32880-2)
Supplement: Supplementary file 6 — Reporting Summary [file 41467_2022_32880_MOESM6_ESM.pdf]

nature portfolio

Corresponding author(s): Heng-Chi Lee
Last updated by author(s): Jun 24, 2022

Reporting Summary

Nature Portfolio wishes to improve the reproducibility of the work that we publish. This form provides structure for consistency and transparency in reporting. For further information on Nature Portfolio policies, see our Editorial Policies and the Editorial Policy Checklist.

Statistics

For all statistical analyses, confirm that the following items are present in the figure legend, table legend, main text, or Methods section.

n/a	Confirmed	
[ ]	[x]	The exact sample size (*n*) for each experimental group/condition, given as a discrete number and unit of measurement
[ ]	[x]	A statement on whether measurements were taken from distinct samples or whether the same sample was measured repeatedly
[ ]	[x]	The statistical test(s) used AND whether they are one- or two-sided<br>*Only common tests should be described solely by name; describe more complex techniques in the Methods section.*
[x]	[ ]	A description of all covariates tested
[x]	[ ]	A description of any assumptions or corrections, such as tests of normality and adjustment for multiple comparisons
[ ]	[x]	A full description of the statistical parameters including central tendency (e.g. means) or other basic estimates (e.g. regression coefficient) AND variation (e.g. standard deviation) or associated estimates of uncertainty (e.g. confidence intervals)
[ ]	[x]	For null hypothesis testing, the test statistic (e.g. *F*, *t*, *r*) with confidence intervals, effect sizes, degrees of freedom and *P* value noted<br>*Give P values as exact values whenever suitable.*
[x]	[ ]	For Bayesian analysis, information on the choice of priors and Markov chain Monte Carlo settings
[x]	[ ]	For hierarchical and complex designs, identification of the appropriate level for tests and full reporting of outcomes
[x]	[ ]	Estimates of effect sizes (e.g. Cohen's *d*, Pearson's *r*), indicating how they were calculated

Our web collection on statistics for biologists contains articles on many of the points above.

Software and code

Policy information about availability of computer code

Data collection	Bio-Rad CFX96 Touch Real-Time PCR Detection System was used for qPCR<br><br>Illumina HiSeq4000 or NovaSeq 6000 were used for sequencing<br><br>Confocal imaging was performed using Zeiss LSM800 Confocal Microscope with 40X or 63X oil objective.<br><br>Q Exactive HF-X mass spectrometer was used for mass spectrometry.

Data analysis	mRNA-seq, small RNA-seq, RIP-seq analysis:<br>- bowtie v1.2.1.1<br>- bowtie2 v2.3.0<br>- cutadapt v1.18<br>- perl v5.24.0 - custom scripts<br>- R v4.0.0 - custom scripts, ggplot2 v3.3.6, reshape2 v1.4.4, ggpubr v0.4.0, dplyr v1.0.9<br>- bedtools v2.27.1<br><br>Image analysis:<br>- ImageJ v1.53q<br><br>Mass spectrometry analysis:<br>- MaxQuant v1.6.1.0

For manuscripts utilizing custom algorithms or software that are central to the research but not yet described in published literature, software must be made available to editors and reviewers. We strongly encourage code deposition in a community repository (e.g. GitHub). See the Nature Portfolio guidelines for submitting code & software for further information.

Data

Policy information about availability of data

All manuscripts must include a data availability statement. This statement should provide the following information, where applicable:

- Accession codes, unique identifiers, or web links for publicly available datasets
- A description of any restrictions on data availability
- For clinical datasets or third party data, please ensure that the statement adheres to our policy

All sequencing data generated in this study (mRNA-seq, small RNA-seq, and RIP-seq) are available at the NCBI SRA database with accession number PRJNA802581 (https://www.ncbi.nlm.nih.gov/bioproject/?term=PRJNA802581). Small RNA-seq from the parn-1 mutant 27 can be found with accession number SRS1021265 (https://www.ncbi.nlm.nih.gov/sra/?term=SRS1021265). The MS proteomics data have been deposited to the ProteomeXchange Consortium via the PRIDE partner repository with the accession number PXD033506 (https://www.ebi.ac.uk/pride/archive/projects/PXD033506). Source data are provided with this paper. All custom scripts are available upon request to the corresponding authors.

Field-specific reporting

Please select the one below that is the best fit for your research. If you are not sure, read the appropriate sections before making your selection.

☒ Life sciences ☐ Behavioural & social sciences ☐ Ecological, evolutionary & environmental sciences

For a reference copy of the document with all sections, see nature.com/documents/nr-reporting-summary-flat.pdf

Life sciences study design

All studies must disclose on these points even when the disclosure is negative.

Sample size	For RNA-seq, small RNA-seq and RIP-seq experiments, at least 5,000 worms were used to obtain sufficient starting RNA material according to the library preparation kit manufacturer's recommendation. For imaging, at least 5 adults, embryos, germlines were used (specifics disclosed in figure legends), determined by intact tissue successfully mounted for imaging. For the piRNA initiation assay, at least 14 worms were used, determined by the number of whole worms successfully mounted for imaging. For piRNA reporter assays, at least 15 worms were used, determined by the number of whole worms successfully mounted for imaging.
Data exclusions	No data were exclueded
Replication	All imaging, RNA-seq, small RNA-seq, and RIP-seq experiments were performed independently at least two times and replicates were compared. No inconsistent results were observed. HRDE-1 IP RIP-seq in glh-1 single mutants was performed only once due to limited starting RNA material.
Randomization	For all sequencing and imaging experiments, control and experimental samples were treated completely in parallel. Experiments were not randomized and controlling for covariates was not necessary for the following reasons. For all imaging experiments, worms were imaged based on genotype and chosen randomly for imaging/processing from stock plates/coverslips. Data was collected from all worms randomly chosen in each condition, and all analysis was done programmatically with perfectly identical conditions across genotypes, thus limiting biases in interpretation based on foreknowledge of the genetic background. For sequencing experiments, data was analyzed programmatically in a robust analysis pipeline and statistical tests were performed across conditions and replicates. Thus, biases in interpretation of single data points were avoided and possible covariates that would result in significant deviation in results across replicates were monitored.
Blinding	Blinding was not possible for most experiments due to phenotypic variation that is obvious under microscopy decisions. When possible, tubes were coded numerically, not by genotype/condition, for sequencing experiments. For smFISH experiments, slides were coded numerically and imaged naively, not based on genotype.

Reporting for specific materials, systems and methods

We require information from authors about some types of materials, experimental systems and methods used in many studies. Here, indicate whether each material, system or method listed is relevant to your study. If you are not sure if a list item applies to your research, read the appropriate section before selecting a response.

Materials & experimental systems

n/a	Involved in the study
☐	☒ Antibodies
☒	☐ Eukaryotic cell lines
☒	☐ Palaeontology and archaeology
☐	☒ Animals and other organisms
☒	☐ Human research participants
☒	☐ Clinical data
☒	☐ Dual use research of concern

Methods

n/a	Involved in the study
☒	☐ ChIP-seq
☒	☐ Flow cytometry
☒	☐ MRI-based neuroimaging

Antibodies

Antibodies used

GFP-Trap Magnetic Agarose (ChromoTek)
Anti-Flag Magnetic Beads (bioLinkedin)
Anti-PRG-1 primary antibody (Batista et al. 2008)
Anti-Rabbit Alexa488 (Jackson Labs Cat. No. 711-547-003)

Validation

GFP-Trap Magnetic Agarose has been validated by Barucci et al. Nature Cell Biology 2020
Anti-Flag Magnetic Beads has been validated by Barucci et al. Nautre Cell Biology 2020
Anti-PRG-1 primary antibody has been validated by Batista et al. 2008
Anti-Rabbit Alexa488 has been validated by Jackson Labs

Animals and other organisms

Policy information about studies involving animals; ARRIVE guidelines recommended for reporting animal research

Laboratory animals

All the data collected for this study derived from hermaphrodite Caenorhabditis elegans nematode culture at the appropriate embryonic or adult stage as described in the methods and figure legends.
The strains used in this study are glh-1(uoc1) I, glh-1DQAD(uoc3) I, glh-1FGGΔ(uoc4) I, glh-1(uoc5) glh-4(gk225) I/hT2 [bli-4(e937) let-?(q782) qIs48] (I;III), deps-1(bn124) I, meg-3(ax3055) meg-4(ax3052) X., and mip-1(uae1) III; mip-2(uae2) V.

Wild animals

No wild animals were used in this study.

Field-collected samples

No samples were collected from the field.

Ethics oversight

No ethical approval was required.

Note that full information on the approval of the study protocol must also be provided in the manuscript.

March 2021
